# 1,4-Naphthoquinones: From Oxidative Damage to Cellular and Inter-Cellular Signaling

**DOI:** 10.3390/molecules190914902

**Published:** 2014-09-17

**Authors:** Lars-Oliver Klotz, Xiaoqing Hou, Claus Jacob

**Affiliations:** 1 Department of Nutrigenomics, Institute of Nutrition, Friedrich-Schiller-University Jena, Dornburger Str. 29, 07743 Jena, Germany; 2 Faculty of Pharmacy and Pharmaceutical Sciences, University of Alberta, 2055 Katz Group Centre for Pharmacy and Health Research, 11361 87Ave, Edmonton, AB T6G 2E1, Canada; 3 Division of Bioorganic Chemistry, School of Pharmacy, Saarland State University, Campus, Building B 2.1., Room 1.13, 66123 Saarbruecken, Germany

**Keywords:** oxidative stress, redox cycling, NQO1, Nrf2, epidermal growth factor receptor, protein tyrosine phosphatases (PTP), gap junction, connexin

## Abstract

Naphthoquinones may cause oxidative stress in exposed cells and, therefore, affect redox signaling. Here, contributions of redox cycling and alkylating properties of quinones (both natural and synthetic, such as plumbagin, juglone, lawsone, menadione, methoxy-naphthoquinones, and others) to cellular and inter-cellular signaling processes are discussed: (i) naphthoquinone-induced Nrf2-dependent modulation of gene expression and its potentially beneficial outcome; (ii) the modulation of receptor tyrosine kinases, such as the epidermal growth factor receptor by naphthoquinones, resulting in altered gap junctional intercellular communication. Generation of reactive oxygen species and modulation of redox signaling are properties of naphthoquinones that render them interesting leads for the development of novel compounds of potential use in various therapeutic settings.

## 1. Introduction: Naphthoquinones as Redox Cyclers and Alkylating Agents

**Figure 1:**
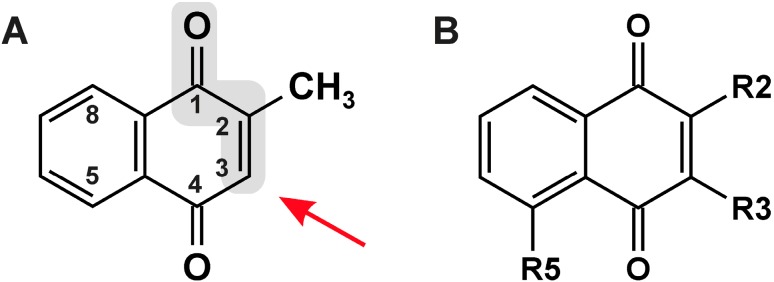
Structures of naphthoquinones. (**A**) Menadione (2-methyl-1,4-naphthoquinone), its α,β-unsaturated carbonyl moiety (gray) and the site of attack of a nucleophile in a Michael-type addition (red arrow); (**B**) Naphthoquinones mentioned in this article (see Table 1 for R2, R3, R5).

Vitamin K assists in coagulation by allowing for the γ-carboxylation of glutamyl residues of proteins involved in the blood clotting cascade—thereby generating calcium chelating moieties. All vitamin K forms (vitamers) are 2-methyl-1,4-naphthoquinone (menadione, also termed vitamin K_3_) derivatives, the most prominent being phylloquinone, which is of plant origin (vitamin K_1_; with a phytyl side chain in position 3, “R3” in Figure 1), and menaquinones (vitamin K_2_; with polyisoprenoid side chains in position 3) of bacterial origin (including intestinal bacteria) [1]. Cycling between the oxidized quinone and the reduced hydroquinone forms of K vitamers is crucial to their biological activity [1]. This quinone/hydroquinone interchange and, more generally, the reducibility of naphthoquinones in biological systems, is also the basis for several biological activities of non-vitamin K-naphthoquinones of natural origin. For example, plumbagin (as found in *Plumbago* sp., e.g., leadwort) or juglone (from certain types of walnut, *Juglans* sp.) are excellent redox cyclers, causing generation of reactive oxygen species (ROS) in cells exposed to these quinones [2,3].

In the case of naphthoquinones, redox cycling represents a cyclic process of reduction of a compound, followed by (aut)-oxidation of the reaction product under concomitant generation of ROS [4]. It requires both suitable reducing equivalents and electron acceptors for oxidation of the reaction product that will cause the formation of ROS. In the case of 1,4-naphthoquinones and mammalian cells, the reduction of these quinones may occur at the expense of NADH or NADPH, as catalyzed by several alternate enzymes. For example, cytochrome P450 reductase would catalyze the simple reduction of the naphthoquinone to the corresponding semiquinone [5], which can, in turn, be oxidized by molecular oxygen. Oxygen is reduced to form superoxide, O_2_^•−^, which will then undergo disproportionation (“dismutation”) to O_2_ and hydrogen peroxide—both with and without facilitation by superoxide dismutases. Alternatively, 1,4-naphthoquinones may undergo a two-electron reduction to the corresponding hydroquinone, which is catalyzed by NAD(P)H:quinone oxidoreductase-1 (NQO-1, DT-diaphorase [6]). Although contributing to xenobiotic metabolism in that hydroquinones can now undergo Phase II metabolism [7] (coupling of the hydroxyl moieties to water-soluble molecules to facilitate elimination), they may also be unstable and be oxidized by oxygen to form the semiquinone and superoxide [7]. This two-faced role of NQO1 in quinone metabolism was recently illustrated in HEK293 cells exposed to menadione [8]. Cells overexpressing NQO1 were more sensitive to menadione than the corresponding control cells, they generated ROS to a higher extent and menadiol (the hydroquinone form of menadione) levels were also high. A concomitant overexpression of UDG-glucuronosyl transferases, however, prevented these effects and rendered cells more resistant [8]. Pro- and anti-oxidant roles of NQO1 in quinone metabolism were also summarized by Cadenas [9].

Naphthoquinones with a free position in conjugation to one of the carbonyls, such as C-3 in menadione (Figure 1), may react with nucleophiles, such as thiols or amines, and form adducts via a so-called Michael addition reaction. Importantly, for such a reaction to occur, the substituent at C-2 has to allow both for access to and sufficient electrophilicity of C-3: lawsone (2-hydroxy-1, 4-naphthoquinone), for example, is a comparatively weakly alkylating agent [2,10]. In contrast, menadione and other naphthoquinones (e.g., #1, 2, 4, Table 1) interact with nucleophiles, such as glutathione, causing significant GSH modification and, hence, depletion in cells exposed to these compounds [2,11,12].

**Table 1:** List of naphthoquinones mentioned in this article (see Figure 1B for positions of R2, R3, R5).

#	R2	R3	R5	Names/Acronyms Used
**1**	H	H	H	1,4-Naphthoquinone
**2**	H	H	OH	Juglone
**3**	CH_3_	H	H	Menadione/MQ
**4**	CH_3_	H	OH	Plumbagin
**5**	OH	H	H	Lawsone
**6**	OH	CH_2_-CH=C(CH_3_)_2_	H	Lapachol
**7**	OCH_3_	H	H	MeONQ
**8**	OCH_3_	OCH_3_	H	DMNQ
**9**	S-CH_2_-CH_2_-OH	S-CH_2_-CH_2_-OH	H	NSC 95397

Interestingly, alkylation and redox cycling may join forces: after addition of glutathione to naphthoquinones—e.g., to C-3 in menadione, to result in 3-glutathionyl-2-methyl-1,4-naphthodiol [13]—the reaction product may undergo autoxidation, generating superoxide and the semiquinone and quinone forms, the latter of which may be reduced back by NQO1 [13]. The oxidation and alkylation of numerous biomolecules was demonstrated for cells exposed to menadione or other naphthoquinones, and appears to be responsible for most of their cytotoxic effects. In fact, menadione-derived compounds have been discussed as anti-tumor drugs (or pro-drugs, as the semiquinone radical formed during activation of menadione by reduction may be regarded as active form) [5].

In summary, many biological effects of 1,4-naphthoquinones may be deduced from, and explained by, their redox cycling and alkylating properties. See Reference [14] for a recent review.

## 2. Naphthoquinone-Induced Intra- and Intercellular Signaling

Redox and alkylating activities of naphthoquinones also correspond to some of their signaling activities. For example, in HaCaT human keratinocytes those quinones that most effectively depleted or oxidized glutathione or generated superoxide were the most active stimulators of ErbB family receptor tyrosine kinases [2]. In addition to quinone-induced ErbB signaling and its consequences, we will briefly discuss the relationship between naphthoquinone effects and modulation of transcription factor Nrf2.

### 2.1. Menadione and “Menadione Reductase”: Naphthoquinones, Nrf2 Signaling and the Expression of Protective Genes

Early names of NQO1 included “vitamin K reductase” or “menadione reductase” owing to the fact that these naphthoquinones are recognized as substrates by the enzyme (see [6] for a historical overview, and [15] for a recent review). Several inducers of NQO1 expression have been identified (for a comprehensive list, see [16]). Talalay and colleagues identified a feature common to many of these inducers, *i.e*., their susceptibility to nucleophilic attack—in fact, many of these compounds are good electrophiles in Michael-type addition reactions [17]. It comes as no surprise, therefore, that naphthoquinones are among these NQO1 inducing compounds. For example, menadione was identified as a NQO1 inducer in murine liver [18].

Induction of NQO1 by these compounds was traced back to the presence of a promoter element called both “antioxidant response element” (ARE) and “electrophile response element” (EpRE) [19]. AREs were found to be present also in the promoter regions of other genes induced by the above compounds, such as those coding for heme oxygenase-1 or for enzymes required for glutathione biosynthesis [20]. Stimulation of ARE-driven transcription is elicited by transcription factor Nrf2, whose accumulation in the nucleus is triggered by conditions that attenuate cytoplasmic Nrf2 degradation. Kelch-like ECH-associated protein 1 (Keap1) sequesters Nrf2 in the cytoplasm and bridges it to a ubiquitin ligase, Cul3, to facilitate proteasomal Nrf2 degradation. Keap1 is a cysteine-rich protein susceptible to oxidation and alkylation, both of which may result in altered Keap1/Nrf2 interaction, decreased Nrf2 degradation and Nrf2 accumulation, followed by Nrf2 nuclear transport (for reviews, see [15,20]). Nrf2, which is required for NQO1 induction by such stimuli as sulforaphane [21], has protective properties in cells exposed to naphthoquinones: menadione, DMNQ (see Table 1) and 1,2-naphthoquinone toxicities to cultured mouse embryonal fibroblasts (MEF) were attenuated significantly by MEF pretreatment with sulforaphane [21,22]. Similarly, toxicity of 1,2-naphthoquinone was elevated in murine hepatocytes deficient in Nrf2 compared to wild type cells, whereas toxicity was lower in Keap1-deficient cells [23]. Moreover, ROS formation induced by menadione was higher [24] and basal DMNQ toxicity was enhanced [21] in Nrf2-deficient MEF. 1,2-naphthoquinone-induced ROS production was elevated in Nrf2-deficient murine hepatocytes, as was the formation of 1,2-naphthoquinone adducts with cellular proteins [23]. Adduct formation was also observed with Keap1 in these cells, followed by activation of Nrf2 and Nrf2-dependent gene expression [23]. Menadione modifies isolated Keap1 [25], weakly stimulates Nrf2 accumulation in murine macrophages [26] and human hepatoma cells [27] and stimulates the expression of Nrf2 target genes in exposed cells [28]. Nevertheless, it appears that menadione-induced Nrf2 target gene expression may occur predominantly via other signaling cascades, as hypothesized for menadione-induced HO-1 expression, which was only slightly diminished in Nrf2-deficient cells [28]. The authors hypothesize that stimulation of transcription factor NF-κB may instead mediate elevation of HO-1 expression by menadione. NF-κB was shown to be stimulated by the quinone, which also appears to contribute to cellular protection against menadione toxicity [29].

In line with the weak effects of menadione on Nrf2 signaling in these models, menadione did not stimulate ARE-dependent signaling in cultured neuronal cells—whereas its 5-hydroxylated derivative, plumbagin, strongly activated Nrf2/ARE signaling [30]. Plumbagin stimulated expression of Nrf2 target genes, including NQO1 and HO-1 in these cells, and preincubation with plumbagin was protective (via stimulation of Nrf2) against peroxide stress or deprivation of glucose or of oxygen. Intravenous application of plumbagin to mice even protected against ischemic stroke in a model for focal cerebral ischemia/reperfusion injury [30]. Plumbagin and another naphthoquinone, 5,8-dihydroxy-1,4-naphthoquinone (naphthazarin, or 8-hydroxy-juglone), also stimulated Nrf2/ARE signaling in HepG2 cells [31,32]. Moreover, naphthazarin enhanced expression of Nrf2 target genes in primary rat neuronal cells, and preincubation with naphthazarin attenuated glutamate toxicity [31].

The roundworm, *Caenorhabditis elegans*, serves as a convenient *in vivo* model for research on metabolic and stress signaling because central signaling pathways are highly conserved from worm to mammalia. Plumbagin elicited an enhanced expression of genes known to be controlled by ARE activation [32]. Like plumbagin, menadione and naphthazarin stimulated an ARE reporter, indicating that they activate signaling by the *C. elegans* ortholog of Nrf2, SKN1. Exposure of *C. elegans* worms to non-toxic concentrations of naphthoquinones, such as juglone, plumbagin and naphthazarin, not only *not* affected *C. elegans* life span, but, rather, resulted in life span *extension* (by some 10%) at low quinone concentrations [32,33], a classical hormetic response to naphthoquinones. Interestingly, this effect was not seen with menadione [32]. Depletion of SKN1 abrogated ARE activation and lifespan extension [32].

In addition to interaction with Keap1, posttranslational modification was described as a means of regulating Nrf2 activity. For example, ERK, JNK and p38 mitogen-activated protein kinases (MAPK) all phosphorylate Nrf2, albeit with only minor contribution to Nrf2 nuclear accumulation and Nrf2-dependent target gene expression [34]. As several naphthoquinones happen to strongly stimulate MAPK (see also Section 2.2), these quinones may elicit Nrf2 signaling by redox cycling and/or alkylation-dependent processes also independently of Keap1 modification.

In summary, some naphthoquinones stimulate Nrf2-dependent activation of ARE-dependent gene expression, followed by enhanced formation of protective proteins, such as enzymes involved in glutathione biosynthesis—and NQO1. This naphthoquinone-induced Nrf2-dependent protective response appears to be highly conserved, as the same signaling cascade is stimulated in roundworms and mammalia.

### 2.2. Stimulation of Receptor Tyrosine Kinase Signaling by Naphthoquinones—Consequences for Gap Junctional Intercellular Communication

Several naphthoquinones are strong stimulators of growth factor receptors of the receptor tyrosine kinase (RTK) family, and of RTK-dependent signaling. For example, exposure to naphthoquinones may elicit tyrosine phosphorylation of the ErbB-family RTKs, the epidermal growth factor (EGF) receptor (EGFR) and the related ErbB2 [11,35,36], or of platelet-derived growth factor receptors (PDGFR) [12]. RTK signaling is triggered in the absence of the natural ligands, *i.e*., through mechanisms independent of growth factor stimulation of the respective receptor. The extent of EGFR and ErbB2 activation was shown to correlate with the capability of the respective naphthoquinones to cause the generation of superoxide and depletion of glutathione (GSH): both were demonstrated in HaCaT human keratinocytes exposed to 1,4-naphthoquinone, menadione, juglone or plumbagin, and all quinones strongly stimulated EGFR and ErbB2 activation and downstream signaling [2] (see Table 2). In contrast, no activation of these receptors was detected in cells exposed to lawsone (the active ingredient of henna) or lapachol (from lapacho tree heartwood and bark) [2]. In addition to significant GSH depletion, slight increases in glutathione disulfide (GSSG) levels were observed in cells exposed to quinones, supporting the notion that redox cycling occurs. Superoxide generated by redox cycling would form H_2_O_2_, whose reduction to water would be catalyzed by glutathione peroxidases and would occur at the expense of GSH (which would be oxidized to GSSG) (see Table 2). Interestingly, EGFR activation by menadione, plumbagin, or juglone was diminished by approximately 20% in cells pretreated with manganese (III) tetrakis (4-benzoic acid) porphyrin (MnTBAP), a superoxide dismutase and catalase-mimetic. This indicates that ROS formation may be involved in EGFR activation, but a larger portion of the activation is likely due to other factors. In line with this notion, EGFR activation by 1,4-naphthoquinone was not attenuated by MnTBAP, implying that, although significant ROS production is detectable in cells exposed to this quinone, stimulation of EGFR signaling is elicited independently of ROS formation. In fact, all of the actively signaling quinones exhibit alkylating activity, as judged by the strong depletion of GSH noted in the presence of these compounds (Table 2).

**Table 2:** Oxidative stress and EGFR/ErbB2 activation in HaCaT cells exposed to naphthoquinones (3–100 µM) for 1 h [2]. LC_50_: concentration with 50% residual cell viability, determined 24 h after a 1 h exposure of cells to quinone; *E*^1^_pH7_: one-electron reduction potential (pH 7), values from References [37,38]. Arrows: direction of change relative to control treatments; ±, activation/no activation.

#	LC_50_ (µM)	DNA Damage	GSH	GSSG	Superoxide Formation	*E*^1^_pH7_ (mV)	Activation of EGFR/ErbB2	Naphthoquinone
**1**	15	N.D.	↓↓	↑	↑	−140	+++	1,4-Naphthoquinone
**2**	6	↑	↓↓	↑	↑	−93	+++	Juglone
**3**	40	↑↑	↓	↑	↑↑↑	−203	++	Menadione
**4**	5	N.D.	↓↓	↑	↑↑	−156	+++	Plumbagin
**5**	>100	→	→	→	↑/→	−415	−	Lawsone
**6**	>100	N.D.	→	→	→		−	Lapachol

Interestingly, RTK signaling may also be impaired by suitable naphthoquinones: PDGFR inhibitors on a naphthoquinone basis were synthesized and described recently, targeting PDGFR but not EGFR signaling in vascular smooth muscle cells. These quinones are 5,8-dimethoxy-1, 4-naphthoquinone derivatives with intermediate-chain alkyl moieties (nonylamino [39], decylamino [40] and undecylsulfonyl [41]) in position 2—quite different from the short-chain derivatives in Table 1.

RTK stimulation results in activation of several signaling cascades emanating from these receptors, including activation of MAPKs. Specifically, ERK-1, ERK-2, and ERK-5 MAPKs (summarized as ERK^MAPK^ from here on), as well as the phosphoinositide 3'-kinase (PI3K)/Akt cascade are stimulated in various cells exposed to naphthoquinones, including menadione [11,35,42], DMNQ [11], NSC 95397 (Table 1) [43,44,45] and others.

**Figure 2:**
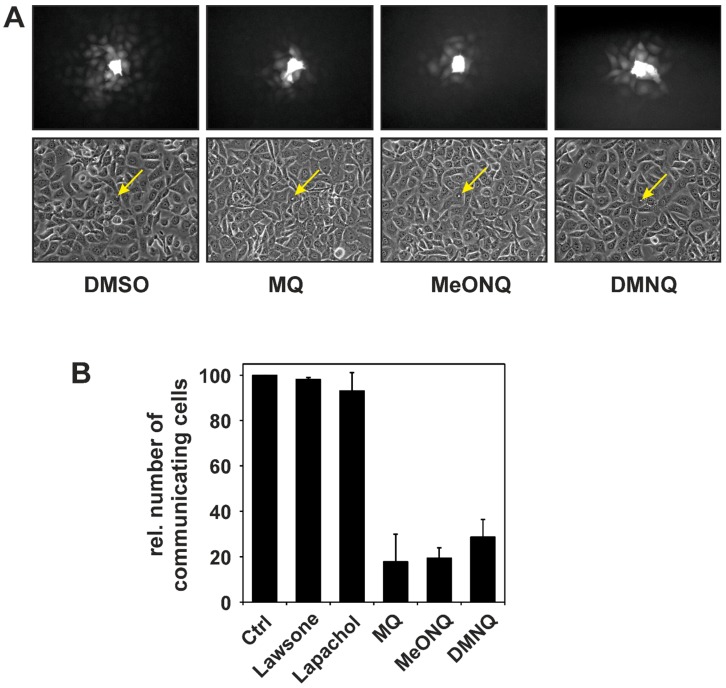
Loss of gap junctional intercellular communication (GJIC) in cells exposed to naphthoquinones. WB-F344 rat liver epithelial cells (a generous gift by Dr. James E. Trosko, Michigan State University, East Lansing, MI, USA) were grown to near confluence, followed by exposure to 10 µM of the given naphthoquinones (or DMSO as vehicle control) in serum-free cell culture medium (DMEM) for 60 min. Cells were washed with PBS and GJIC was determined by microinjecting the fluorescent dye Lucifer Yellow CH into selected cells as described previously [55,56]. One minute after injection, fluorescent cells surrounding the cells loaded with dye were counted and taken as a measure of GJIC. (**A**) Representative fluorescent and phase contrast images taken after microinjection of LY into the cell indicated by a yellow arrow; (**B**) 3 to 10 individual cells were loaded with dye per dish and means of the numbers of fluorescent neighboring cells were calculated. Data are means of three independent experiments performed in duplicate ± S.D. (lapachol, lawsone: *n* = 2, ±min/max).

Stimulation of these cascades affects gene expression. For example, transcription factor AP-1 component proteins are phosphorylated by ERK^MAPK^, JNK^MAPK^ and p38^MAPK^ [46]. In addition to transcription factors, MAPK have multiple other substrates modulating cellular processes such as proliferation and stress response (for a recent review, see [46]). MAPK also regulate inter-cellular communication via gap junctions. Gap junctions are clusters of channels between neighboring cells, essentially connecting their cytoplasms. These channels are composed of connexin molecules that are arranged in two connexin-hexamers that form hemi-channels in the cell membranes of each of the adjacent cells. Gap junctional channels allow for a controlled diffusion of compounds of low molecular mass (up to approximately 1 kDa) between cells (*i.e*., gap junctional intercellular communication, GJIC). Such diffusible compounds include nutrients, such as glucose, signaling molecules and ions, such as cAMP or Ca^2+^, and even oligonucleotides [47,48]. The most prominently expressed of all 21 known human connexins is connexin-43 (Cx43) [47], and gap junctional channels composed of Cx43 are known to be regulated by posttranslational modification of Cx43, such as by phosphorylation [49,50]. Next to protein kinase C, ERK^MAPK^ were among the first kinases identified as Cx43 kinases [51,52]. Cx43 phosphorylation by ERK^MAPK^ was demonstrated to result in lowered conductivity of Cx43 gap junctional channels [52]. Menadione was tested for modulation of GJIC in rat liver epithelial cells. Indeed, exposure of the cells to menadione caused rapid decrease in GJIC, as determined by a dye transfer assay, *i.e*., by microinjection of a non-membrane-permeant fluorescent dye into single cells and assessing the extent of its diffusion into neighboring cells. Numbers of cells showing up as fluorescent after a set period of time are compared and taken as a measure of intercellular communication via gap junctions (see Figure 2A for an example). At subtoxic menadione concentrations, the decrease of GJIC persisted for several hours (unpublished data). Loss of GJIC induced by menadione coincided with Cx43 phosphorylation at sites previously shown to be phosphorylated by ERK^MAPK^ [11,35]. Inhibitors of MAPK/ERK kinases (MEK), the kinases catalyzing the phosphorylation and activation of ERK^MAPK^, attenuated both loss of GJIC and Cx43 phosphorylation. Furthermore, menadione-induced ERK activation as well as loss of GJIC were blocked by inhibitors of EGFR tyrosine kinase activity, pointing to a direct link between quinone-induced activation of EGFR, further on to MEK and to ERK^MAPK^ activation, as well as ERK-dependent Cx43 phosphorylation, resulting in loss of GJIC [11,12]. Other naphthoquinones behaved similarly, in that EGFR-dependent signaling to ERK^MAPK^ was stimulated, followed by ERK^MAPK^-dependent Cx43 phosphorylation, including DMNQ [11] or NSC 95397 [43]. Interestingly, other quinones, such as the anthraquinone derivative doxorubicin, also caused EGFR/ERK-dependent downregulation of GJIC [53].

Some of the quinones listed in Table 1 and Table 2 were never tested for their effects on GJIC, so we exposed WB-F344 rat liver epithelial cells to lawsone, lapachol (*i.e*., the quinones with lowest activities in terms of inducing oxidative stress and stimulating EGFR—see Table 2), menadione as well as MeONQ and DMNQ. The latter two were used to answer the question whether a single change in position 3 (*i.e*., the presence or absence of a methoxy-moiety), which will alter the quinone also with respect to its alkylation and redox cycling properties, changes the cellular response to the quinone in terms of GJIC. While DMNQ is a mere redox-cycling naphthoquinone, MeONQ, like menadione, potently depletes GSH from red blood cells, indicating that, in addition to redox cycling, it may also alkylate thiols [54]. In line with its low activities in HaCaT cells (Table 2) lawsone did not affect erythrocyte GSH levels in the same study, unless glutathione recycling itself was impaired—pointing to a weak oxidative activity of lawsone, which does not affect cellular GSH levels under normal conditions [54]. Neither lawsone nor lapachol significantly affected GJIC in rat liver epithelial cells at the concentrations employed, whereas menadione and both MeONQ and DMNQ caused a significant loss of GJIC in exposed cells (Figure 2). In essence, therefore, the additional methoxy moiety in MeONQ *vs.* DMNQ did not significantly alter the quinone’s impact on GJIC. In line with previous data [11] it appears to be of minor importance whether a quinone affects cellular processes primarily via redox cycling or by alkylation—both appear to be recognized by the cell as stressful stimuli, resulting in stress signaling and modulation of GJIC.

**Figure 3:**
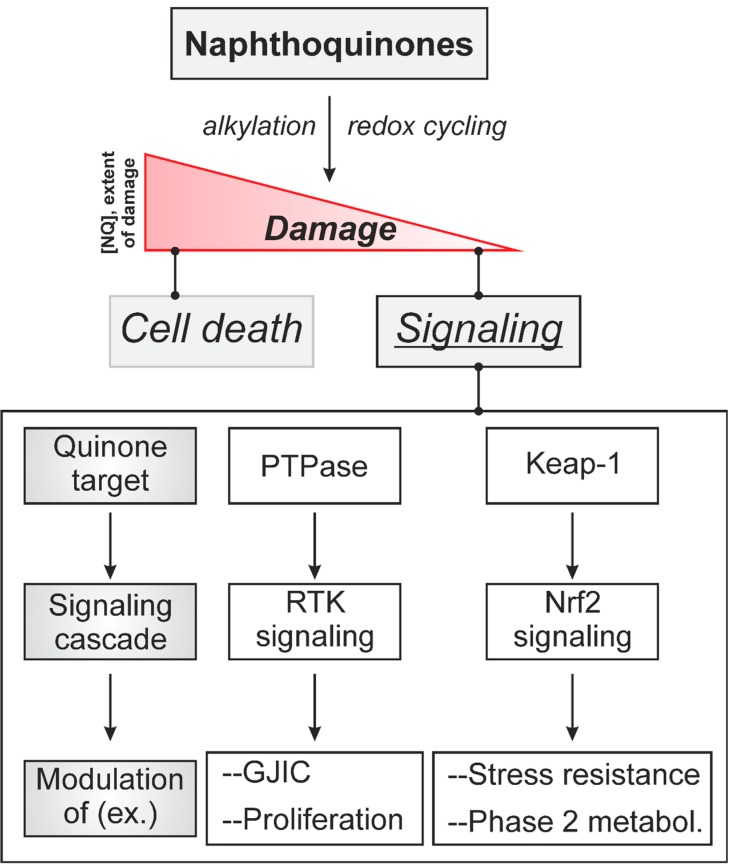
Alkylation and redox cycling as initiators of naphthoquinone-induced signaling. Naphthoquinones (NQ), via oxidation or alkylation of cellular target structures, cause damage that may result in cell death. Stimulation of signaling cascades is triggered already at lower concentrations of NQ (with less extensive damage elicited). The figure provides examples of primary NQ target structures mentioned in the text, signaling cascades stimulated and biological effects elicited. PTPase—protein tyrosine phosphatase; RTK—receptor tyrosine kinase; GJIC—gap junctional intercellular communication.

In conclusion, naphthoquinones—both alkylating and redox cycling—stimulate RTK signaling which, via stimulation of ERK^MAPK^ and Cx43 phosphorylation, affects inter-cellular communication in addition to intracellular signaling responses (Figure 3).

In a tissue with several layers of communicating cells, this would imply that quinones may indirectly affect cells in layers that have not directly seen the quinone in the first place (“bystander” cells).

Whether naphthoquinones affect GJIC in ways other than through connexin phosphorylation, remains to be determined—it is conceivable that stress-responsive mechanisms of posttranscriptional regulation of connexin levels [57] in cells are altered by these compounds. For example, doxorubicin-induced attenuation of GJIC in rat liver epithelial cells was shown to be modulated by the stress-responsive RNA binding protein HuR [58].

## 3. Protein Tyrosine Phosphatases as Targets in Naphthoquinone-Induced Signaling

**Figure 4:**
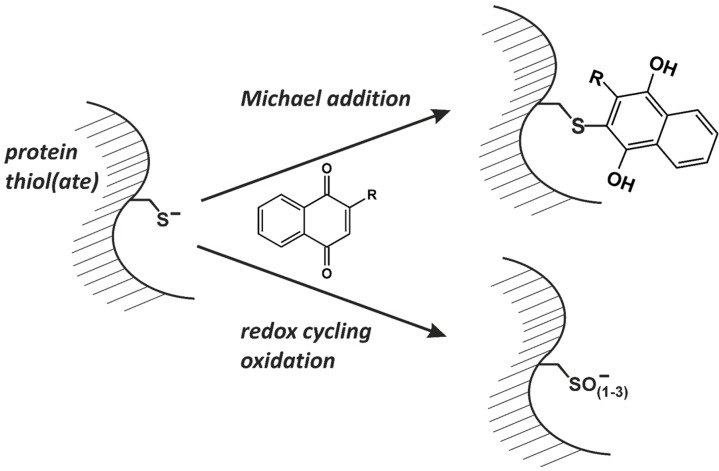
Potential mechanisms for naphthoquinones to cause covalent modification of target proteins. The cysteine thiolate shown can be oxidized by ROS generated through redox cycling to form the corresponding cysteine sulfenate, sulfinate, or sulfonate, and ultimately also a disulfide.

Menadione was demonstrated to inhibit protein tyrosine phosphatase(s) dephosphorylating activated EGFR and ErbB2 [11,36]—suggesting that a mechanism of action of menadione to activate ErbB receptor tyrosine kinases is through PTPase inactivation (Figure 3). Most PTPases are exquisitely sensitive to oxidants and alkylating agents due to their active site cysteine being present in its deprotonated (thiolate) form under physiological conditions [59]. Therefore, several possible mechanisms of PTPase inhibition by naphthoquinones were discussed, including alkylation, oxidation or blockade of the active site, as well as allosteric interactions (Figure 4).

Menadione is capable of alkylating nucleophilic moieties in cells, including thiols [2,11], and of redox cycling [2]. Both, a strong alkylator (*p*-benzoquinone) and an exclusive redox cycler, DMNQ, also stimulated EGFR and EGFR-dependent signaling, yet only for menadione was an inhibition of a PTPase controlling the EGFR detectable [11]. Whilst an inhibitor of NQO-1, dicumarol, blocked DMNQ-induced ERK activation, menadione effects were not impaired by this inhibitor [35].

To test for the inhibitory effects of naphthoquinones in the absence of redox cycling-fostering conditions, isolated PTPase PTP-1B [36], a PTPase regulating the EGFR, and CD45 [35] were assayed for inhibition by naphthoquinones. Menadione inhibited isolated PTPase even under those conditions, suggesting that alkylating properties of this compound might prevail. Although never demonstrated for menadione, this hypothesis is supported by findings on a related naphthoquinone: 1,2-naphthoquinone was demonstrated to inhibit PTP-1B by Michael addition to a non-active site cysteine residue (Cys-121), as well as, to a lesser extent, a non-active site histidine (His-25) [60]. Cys-121 is discussed by the authors as being in close proximity to the active site and therefore able to relay any molecular changes to the enzyme’s active site structure [60].

Interestingly, non-alkylating DMNQ also inhibited isolated PTP-1B, *i.e*., under non-redox cycling conditions—suggesting that non-alkylating, non-oxidative inhibition of PTPases by naphthoquinones is feasible as well. In line with this, NSC 95397 (Table 1), an inhibitor of Cdc25 phosphatases, turned out to be a very potent inhibitor of PTP-1B, as well [36], indicating that naphthoquinones are capable of interacting with the active sites of suitable target enzymes even under non-redox cycling, non-alkylating conditions.

## 4. Conclusions: Naphthoquinones—Useful in a Clinical Setting?

Several quinoid compounds are in use clinically, including chemotherapeutic anthraquinones such as doxo- and daunorubicin, and the mitomycins. Apart from the obvious use of K vitamers to antagonize vitamin K deficiencies (both primary and secondary, e.g., from exposure to vitamin K antagonists), however, simple naphthoquinones as described in this article are mostly “potentials”—*i.e*., they are being investigated with respect to their use in cancer chemotherapy and numerous other therapeutic applications. For an exhaustive treatise on such potential fields of interest for plumbagin, see [61]. Here, three examples of “potential” future fields of application of naphthoquinones will be provided.

### 4.1. Naphthoquinones and Cancer Cells: “Death by ROS”

**Figure 5:**
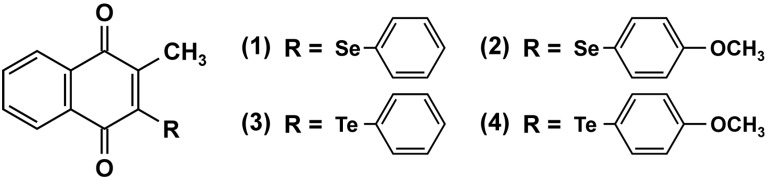
Selenium- and tellurium-containing menadione-derivatives developed by the group of Claus Jacob as multifunctional redox catalysts [65].

The reasoning frequently put forward for research on the use of quinones in cancer therapy is that cancer cells are more susceptible to oxidative damage than their non-cancerous counterparts are: their already elevated levels of ROS [62] would make it easier to drive them “over the top”, *i.e*., towards cell death, by forcing additional ROS upon these cells. For example, one approach successfully tested in cell culture and in mice was the overexpression of manganese superoxide dismutase to catalyze the dismutation of superoxide to hydrogen peroxide—combined with an impairment of peroxide removal, e.g., through drugs interfering with glutathione metabolism [63,64]. Naphthoquinones, therefore, as convenient (and cheap) generators of oxidative stress, might be helpful in this approach. Fry *et al*., synthesized a set of multifunctional selenium- and tellurium-containing naphthoquinones [65]. These menadione derivatives (Figure 5) were intended to damage suitable cancerous cells by generating ROS not only through redox cycling, but also by using the elevated ROS levels in cancer cells to direct them towards cellular biomolecules, *i.e*., to facilitate oxidative damage. The chalcogen added in position 3 would be oxidized by ROS, but the resulting selenoxide or telluroxide would subsequently be reduced easily by cellular components, such as thiols, resulting in the recycling of the chalcogen and the widespread oxidation of cellular thiols and proteins of the “cellular thiolstat” [65]. Exposure to these quinones elicited EGFR-ERK^MAPK^ signaling in rat liver epithelial cells. Both seleno- and telluro-menadiones strongly stimulated ERK^MAPK^ in rat liver epithelial cells, and activation of ERK^MAPK^ was via stimulation of EGFR signaling. These compounds elicited EGFR tyrosine phosphorylation, and inhibition of EGFR, as well as MEK1 and MEK2 abrogated ERK^MAPK^ activation [66]. Although it is likely that stimulation of EGFR-ERK^MAPK^ signaling by multifunctional naphthoquinones will result in a loss of GJIC, this has not been tested so far.

### 4.2. Naphthoquinones and PTPase Inhibition

As described above, naphthoquinones may inhibit PTPases in many ways, and depending on the identity of the PTPase targeted this may affect cellular proliferation. For example, a ligand-independent stimulation of EGFR would be regarded pro-proliferative, owing to the crucial role of this receptor in triggering cell division processes. If, however, the PTPase were to be required for a crucial phase in proliferation to proceed, such as during cell cycle phase transitions, the inhibition of this PTPase may be anti-proliferative. The naphthoquinone NSC 95397 was identified as a selective and potent inhibitor of Cdc25 phosphatases [44]. Cdc25 dual-specificity phosphatases exist in three forms in mammalian cells, Cdc25A-C, and regulate the progression of the cell cycle at the G1-S (Cdc25A) and the G2-M (all Cdc25 forms) transitions [67]. Exposure of different types of cells to NSC 95397, therefore, resulted in cell cycle arrest in G1 [43] or G2 [44] phase and cell death. Whether or not the NSC 95397 pharmacophore will be of use in the development of Cdc25-targeting anti-cancer drugs remains to be seen.

## 4.3. Naphthoquinones and EGFR Activation—Alleviation of Side Effects?

Mutations leading to an overactivity of EGFR (for example through overexpression or generation of a dominant-active mutant) as well as other ErbB family members frequently occur in the genomes of cancerous tissues (see [68] for review). Accordingly, pharmacological inhibitors were and still are being developed to target the EGFR. Both humanized antibodies and kinase inhibitory molecules (low molecular mass inhibitors) are approved for certain cancer therapies [69,70]. Unfortunately, side effects of an EGFR-targeting therapy frequently include severe dermatological phenomena [71,72].

As described above, EGFR activation by naphthoquinones may occur independently of a ligand (such as EGF), and indirectly through targeting a PTPase (*i.e*., through inactivation of a regulatory protein) [11]. From these data, it was inferred that PTPase inhibition might be able to counteract clinically employed EGFR inhibitors by blocking receptor dephosphorylation and, thereby, relieve skin toxicity that was due to EGFR inhibition [73]. Clinical trials are currently underway that investigate the topical use of menadione to alleviate skin toxicities resulting from EGFR inhibitor therapies (ClinicalTrials.gov identifier: NCT01393821).
